# Mesoscopic Patterns of Neural Activity Support Songbird Cortical Sequences

**DOI:** 10.1371/journal.pbio.1002158

**Published:** 2015-06-03

**Authors:** Jeffrey E. Markowitz, William A. Liberti, Grigori Guitchounts, Tarciso Velho, Carlos Lois, Timothy J. Gardner

**Affiliations:** 1 Department of Cognitive and Neural Systems, Boston University, Boston, Massachusetts, United States of America; 2 Department of Biology, Boston University, Boston, Massachusetts, United States of America; 3 Program in Neuroscience, Harvard University, Boston, Massachusetts, United States of America; 4 Department of Neurobiology, University of Massachusetts Medical School, Worcester, Massachusetts, United States of America; 5 Department of Biomedical Engineering, Boston University, Boston, Massachusetts, United States of America; University of Minnesota, UNITED STATES

## Abstract

Time-locked sequences of neural activity can be found throughout the vertebrate forebrain in various species and behavioral contexts. From “time cells” in the hippocampus of rodents to cortical activity controlling movement, temporal sequence generation is integral to many forms of learned behavior. However, the mechanisms underlying sequence generation are not well known. Here, we describe a spatial and temporal organization of the songbird premotor cortical microcircuit that supports sparse sequences of neural activity. Multi-channel electrophysiology and calcium imaging reveal that neural activity in premotor cortex is correlated with a length scale of 100 µm. Within this length scale, basal-ganglia–projecting excitatory neurons, on average, fire at a specific phase of a local 30 Hz network rhythm. These results show that premotor cortical activity is inhomogeneous in time and space, and that a mesoscopic dynamical pattern underlies the generation of the neural sequences controlling song.

## Abstract 2

Cortical premotor activity during singing behavior in songbirds is correlated spatiotemporally over a length scale of 100 µm, with neurons and interneurons firing at opposite phases of a 30 Hz network rhythm. Read the Synopsis.

## Author Summary

“Time cells” can be found throughout the vertebrate forebrain in various species and behavioral contexts. These neurons fire sparsely at precise times during a stereotyped behavior; however, how a neural circuit supports this remarkable property is not known. Here, we describe that the premotor neuronal circuit that is required in birds for singing—“HVC”—is organized in a spatial and temporal manner that supports sparse sequences of neural activity. During song, one class of principal neuron fires during a specific phase of a 30 Hz network rhythm. Fluorescent imaging using head-mounted microscopes reveals that calcium activity in nearby principal neurons is correlated in time with a length scale of 100 μm. Thus, variations in the phase or precise timing of the network rhythm across HVC can provide a mechanism for coordinating the activity of principal neurons. This observation suggests that the timing of principal cells is supported by a mesoscopic spatiotemporal pattern of neural activity.

## Introduction

For many sensory systems, features of sensory inputs are represented topographically in the brain. Examples include orientation tuning in visual cortex, pitch tuning in auditory cortex, and the representation of touch in somatosensory cortex. Motor cortical circuits also contain maps for distinct muscle groups and preferred direction of movement [1,2], but it is not known whether higher-order features, such as time or serial order in a movement sequence, are represented topographically. The specific question of how time is mapped in space is particularly tractable to address in the songbird premotor nucleus HVC (used as a proper name), which is known for producing extremely precise, learned temporal sequences. Projection neurons in HVC fire sparse bursts of action potentials that are precisely aligned to singing. One subtype (HVC_RA_) drives downstream areas controlling the vocal organ [3], while another (HVC_X_) projects to the basal ganglia, which is hypothesized to guide exploratory trial and error learning [4]. These cells can be compared with a larger class of “time cells,” observed in rodents, that fire at specific moments during a stereotyped behavior and may be involved in motor sequence generation, navigational planning, or episodic memory [5–10]. In addition to these sparsely firing cells, HVC contains inhibitory interneurons that produce dense, stereotyped firing patterns during singing [11].

Neither the interaction between inhibitory neurons and projection neurons nor the spatiotemporal organization of HVC activity have been observed in singing birds. In spite of decades of detailed electrophysiology, these aspects of song coding have been impossible to address since imaging and high channel count physiology in singing birds have been technically infeasible until recently. In parallel with these experimental limitations, dynamical models of the HVC network have generally been framed in terms of the neuronal connectivity that supports sequence generation, but the spatial organization of cellular activity has been considered irrelevant [6,12]. However, a number of experimental studies have suggested that HVC activity is spatially organized. Multi-unit activity in HVC is strongly modulated on a 30–100 ms timescale in singing birds [13,14], which could suggest correlated activity in nearby cells. In anesthetized animals, calcium imaging has revealed that spontaneous firing in HVC_X_ neurons is correlated over space (λ = 263 μm) [15]. Retrograde tracer injections and stimulation experiments in HVC show a high degree of lateral connectivity along the rostrocaudal axis [16,17]. Newborn HVC_RA_ projection neurons form soma–soma contacts with existing HVC_X_ neurons at the moment of integration into the song circuit [18]. Finally, multi-electrode recordings in anesthetized birds demonstrated a spatial anisotropy in spike–spike coherence between different cell types: projection neurons were correlated in the rostrocaudal direction and inhibitory neurons in the mediolateral direction [19]. Taken together, these studies suggest that HVC may represent time with some degree of spatial organization, but direct measurements in singing birds are lacking.

In addition to ignoring spatial correlations, models of HVC have largely ignored temporal correlations between inhibition and excitation [6,12] (though some exceptions can be found [20,21]). Yet, excitatory and inhibitory neurons are known to be interconnected in HVC [22,23], HVC_X_ neurons fire bursts of rebound spikes after inhibition in slice [24], and HVC_X_ spiking is anti-correlated with inhibitory neurons in anesthetized songbirds [25]. These observed correlations do not necessarily apply to the singing state. Indeed, in singing birds there appears to be a high diversity of interneuron firing patterns [26], and this diversity suggests that net inhibition on any given projection neuron could be a tonic signal that is relatively unmodulated in time. However, if inhibitory inputs to projection neurons contain synchronous bursts or pauses in firing, the impact on projection neuron firing times could be profound. As a result, the question of how excitatory and inhibitory cells interact in HVC is intrinsically related to the question of whether or not there is a spatial organization or synchrony among neighboring cells in the HVC microcircuit.

**Fig 1:**
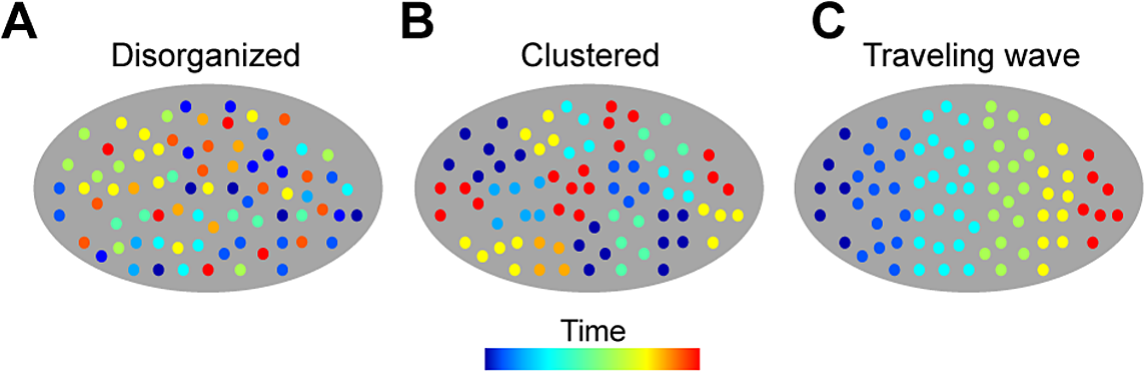
Three hypothetical models for the spatiotemporal organization of the song premotor code in HVC. The spatial organization of neural activity in HVC in singing birds is unknown. The geometry of neural activity could be described by three schematics that form a continuum: **a,** a random, disorganized geometry [8]; **b,** functional clustering (i.e., nearby cells code for similar elements) with a characteristic length scale [27]; and **c,** traveling waves [28].

Here we provide what is, to our knowledge, the first experimental study of how ensemble activity in HVC is organized in space and time in singing zebra finches. To do this, we developed new methods for multi-channel electrophysiology and calcium imaging in singing birds. We address activity patterns of projection neurons and inhibitory interneurons, and investigate the rules that relate the two cell classes together. First, we frame the question of how HVC activity is spatiotemporally organized in terms of three simple models that have been championed in various species (**Fig 1**) [8,27,28]. The schematic models range from a random (**Fig 1A**) to a globally organized geometry (**Fig 1C**). Through calcium imaging and multi-electrode recordings in singing birds, we find that activity patterns of excitatory and inhibitory neurons are spatially clustered (resembling **Fig 1B**), and that two cell types, HVC_X_ projection neurons and HVC interneurons, fire in alternating phases of a local 30 Hz rhythm. Our results show that there is a fundamental mesoscopic length scale and timescale in the premotor cortical area HVC. Similar length scales have recently been reported in calcium imaging of cortex in behaving rodents [27,29,30], and similar timescales have been implicated in primate motor control [28,31,32] and in human speech [33], suggesting that these may be core properties of motor cortical microcircuits across species.

## Results

### The Representation of Time in HVC Projection Neurons Is Spatially Correlated over a 100 μm Length Scale

**Fig 2:**
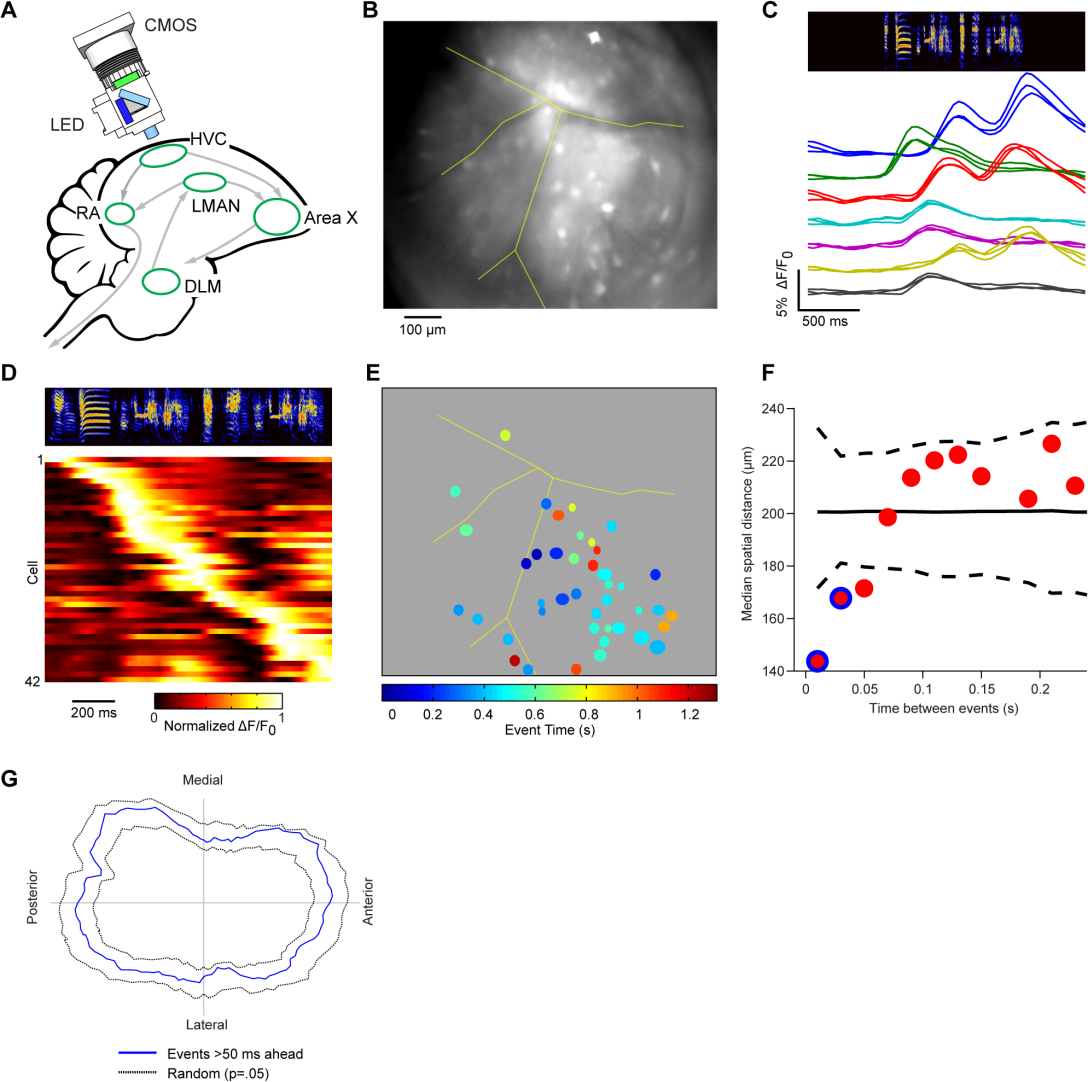
Calcium imaging of HVC projection neurons in awake, behaving birds. **a,** Schematic of the zebra finch brain, showing a simplified song circuit and miniature fluorescence microscope [34]. **b,** Field of view, showing a mean fluorescence image of HVC (blood vessels are traced in yellow). **c**, Several example regions of interest (ROIs) from three trials during a single day of imaging in the same bird, aligned to song (top, spectrogram of example song). **d,** Peak ΔF/F_0_-normalized trial-averaged activity from all song-related neurons from one animal, sorted from earliest to latest peak. **e,** ROIs are colored to represent the time of cell firing within song, defined by the time of 50% rise in the onset of the calcium transient (see **Materials and Methods**). **f**, The median distance between ROIs is shown as a function of the time between their respective calcium events (*n* = 2,230 ROI pairs). This reveals that co-active ROIs (difference between event times <50 ms) are significantly closer to each other than if event times were randomly distributed across HVC (*p* = 0 and *p* =. 038 for the first two points respectively, bootstrap test, Bonferonni corrected). Dotted lines indicate the 95% confidence interval for the null model (see **Materials and Methods**), and the solid line indicates the median. Blue highlighting indicates *p* <.05. **g,** Histogram of relative distances between each ROI and all ROIs with calcium events >50 ms ahead (*n* = 1,710 ROI pairs). The distances are not significantly concentrated relative to the null model described in **f** (*p* =. 6924, bootstrap test). The 95% confidence interval for the histogram of the null model is indicated by dashed lines. The asymmetry of the null model is a consequence of injecting virus at multiple sites distributed along the anterior-posterior (AP) axis of HVC. (The population of recorded cells was presumably elongated in the AP axis as a result.) See also **S1 Fig.**

To examine the spatial organization of calcium activity in HVC, we employed head-mounted fluorescence microscopes (**Fig 2A**) to image the genetically encoded calcium indicator GCaMP6s in freely behaving, singing, adult male zebra finches (*n* = 9 birds). The field of view for these recordings included a large fraction of the surface of HVC, but the lentiviral infections were sparse, limiting our results to observations of *n* = 167 cells across these nine birds (**Fig 2B**, see **Materials and Methods** and **S1 Movie**).

GCaMP6s provided robust calcium signals in single neurons. The observed calcium transients were time-locked and temporally sparse, consistent with the ultra-sparse, time-locked firing patterns reported previously in electrophysiological recordings [3] (**Fig 2B–2D**), and also consistent with evidence that the high-frequency bursts of projection neurons ride on top of calcium plateau potentials [6], but we make no claim that the calcium and electrophysiological signals are equivalent. New technical developments will be needed to directly measure the correspondence between action potentials and calcium in GCaMP6s-infected cells in freely moving birds.

Within this population of sparse firing projection neurons, we found strong spatial correlations in the calcium activity of neighboring neurons (**Fig 2E and 2F**). The median separation between co-active cells (calcium events times separated by less than 20 ms) was 143 μm (119–183 μm, 95% bootstrap confidence interval). Cells that fired at times separated by more than 50 ms showed no spatial correlations with one another (*p* >. 05, bootstrap test; see **Materials and Methods**). These observations were based on the time between sparse calcium events rather than the similarity of the full ΔF/F_0_ time series (e.g., cross-correlation), and are therefore robust to contamination from out-of-plane fluorescence or neuropil (see **Materials and Methods**).

Tests for a traveling wave structure in the data revealed no trend across animals along either the mediolateral or anterior-posterior axis **(** *p* =. 6924, bootstrap test; **Fig 2G)**. In contrast to a geometrically organized traveling wave (**Fig 1C**) [28], the spatial correlations in HVC calcium activity appear to be characterized by a patchwork of domains (**Fig 1B**) organized in stereotyped sequences. These spatiotemporal “domain sequences” do not contain any global geometric organization that we have yet detected. However, we did find a bias for co-active cells to be clustered along the anterior-posterior axis (**S1 Fig**), similar to previous anatomical [16,17] and physiological [19] observations in anesthetized birds.

### Inhibitory Neuron Activity Is Reflected in a 30 Hz Local Field Potential, and This Field Potential Is Also Correlated over a 100 μm Length Scale

We next asked whether other measures of activity in HVC are correlated over a length scale comparable to the correlation length measured in the calcium activity. Simultaneous electrophysiological recordings of interneurons separated by defined spatial distances is not currently feasible in HVC, and the timescale of the calcium indicator used here could not resolve the fast interneuron firing patterns previously described in HVC. Faced with these limitations, we turned to the local field potential (LFP), which can reflect synchronous ensemble neural activity over roughly 100 μm [35,36], though the exact volume of activity represented by the LFP is under debate [37]. In some cases, the LFP also carries timing information about synchronous inhibition [38,39]. To record both LFPs and multiple single-units, we developed an ultra-small carbon fiber electrode array capable of recording 16 channels of LFP and spiking data in singing birds [11]. We recorded 65 putative interneurons, 19 projection neurons, and 268 distinct LFP sites from HVC in adult male zebra finches (*n* = 27 birds).

**Fig 3:**
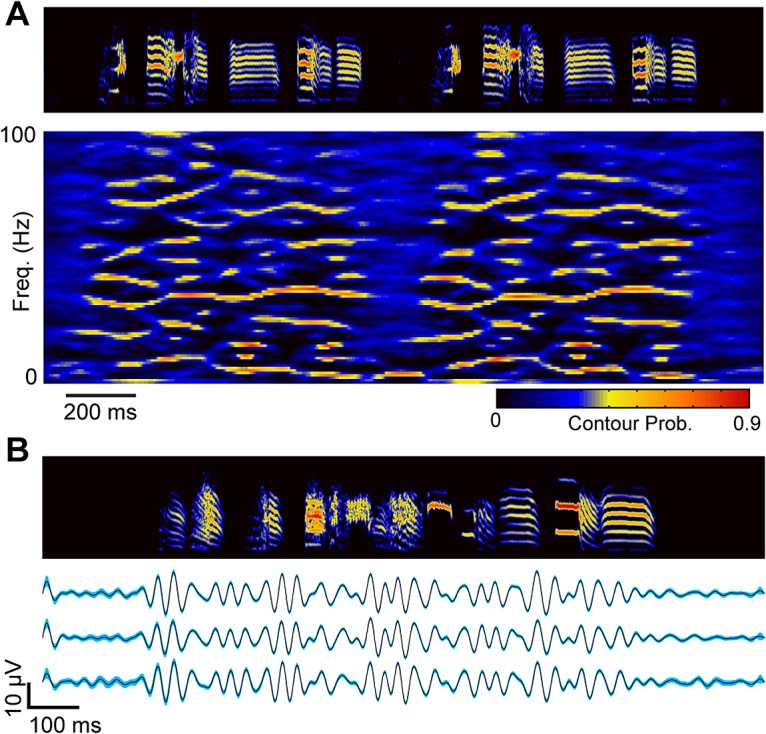
Stereotyped network activity during vocal production in songbird premotor cortex area HVC. **a,** Spectral density images [40] reveal the time-frequency structure in the LFP during singing (*n* = 64 song-aligned trials. No time warping was applied). In a spectral density image, color indicates the probability density of time-frequency structure (see **Materials and Methods**). Reliable time-frequency structure emerges as the bird begins to sing and disappears during the short gap between song motifs. **b,** Trial-averaged band-passed LFPs (25–35 Hz) from a single electrode on three consecutive days (*n* = 130 trials for the top trace, and *n* = 200 trials for the middle and bottom traces). Shading indicates 99% bootstrap confidence interval. See also **S2 Fig.**

**Fig 4:**
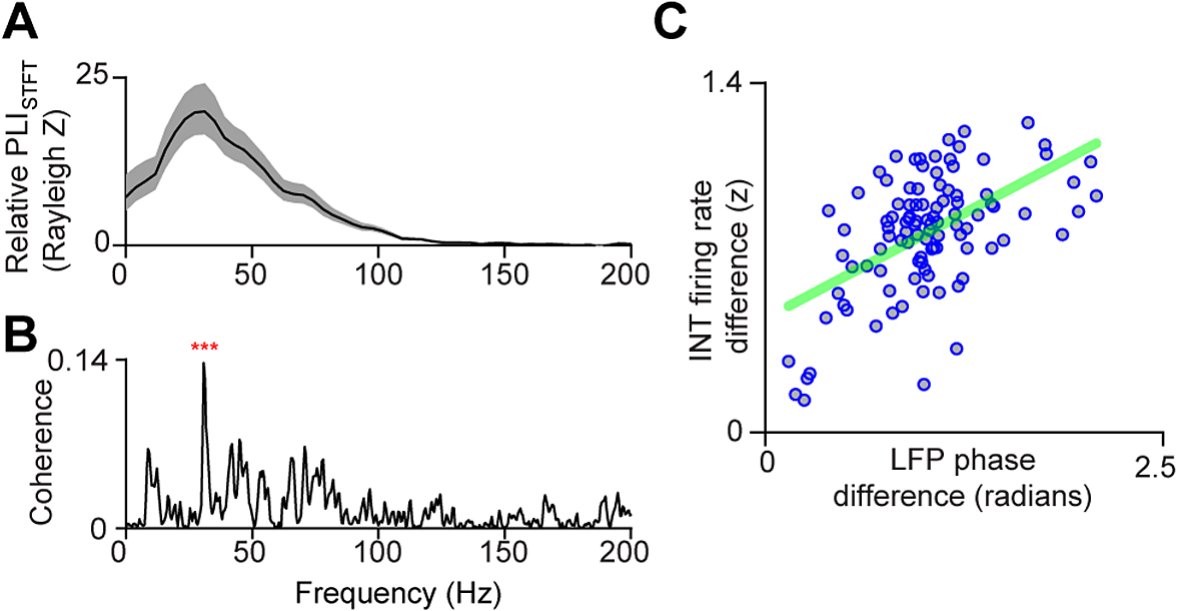
The phase of the 25–35 Hz LFP can be used to study the spatial structure of inhibition. **a,** The average change in phase locking for all birds (PLI_STFT_, see **Materials and Methods**) between song and awake quiescence indicates that the most stereotyped frequency band in the LFP is centered on 30 Hz. **b,** The magnitude squared coherence between LFPs and interneurons (*n* = 17, see **Materials and Methods**) is highly significant at 30 Hz (*p* = 0, bootstrap test, Bonferonni corrected). **c,** For a given pair of recording sites (*n* = 79), phase shifts in the 25–35 Hz LFP predict interneuron firing pattern distance (r =. 48, *p* = 8.5e-6, Pearson correlation coefficient). See also **S3 Fig.**

To date, the LFP has not been described in HVC in singing birds. We found that stereotyped LFP structure appears in the 10–100 Hz frequency range as song begins and rapidly disappears during inter-motif gaps in sound and at the end of song (**Fig 3A**, see **S2 Fig** for single trial examples). In **Fig 3B,** we show the stability of the trial-averaged LFP in the 25–35 Hz frequency band. This particular frequency band is the most phase-locked to song behavior (**Fig 4A** and **S3C Fig**). In support of the importance of the 30 Hz frequency band in the LFP, a strong correlation was found between inhibitory neuron firing times and LFP phase at 30 Hz (**Fig 4B**; cross-spectrum analysis, *p* = 0, bootstrap test). When two spatially separated electrodes in HVC show similar LFP patterns in this 25–35 Hz band, their local interneuron activity is also highly correlated (**Fig 4C**; r =. 48, *p* = 8.5e-6, Pearson correlation coefficient). Hence, the 25–35 Hz LFP can be used as a surrogate to study the spatial structure of interneuron firing.

**Fig 5:**
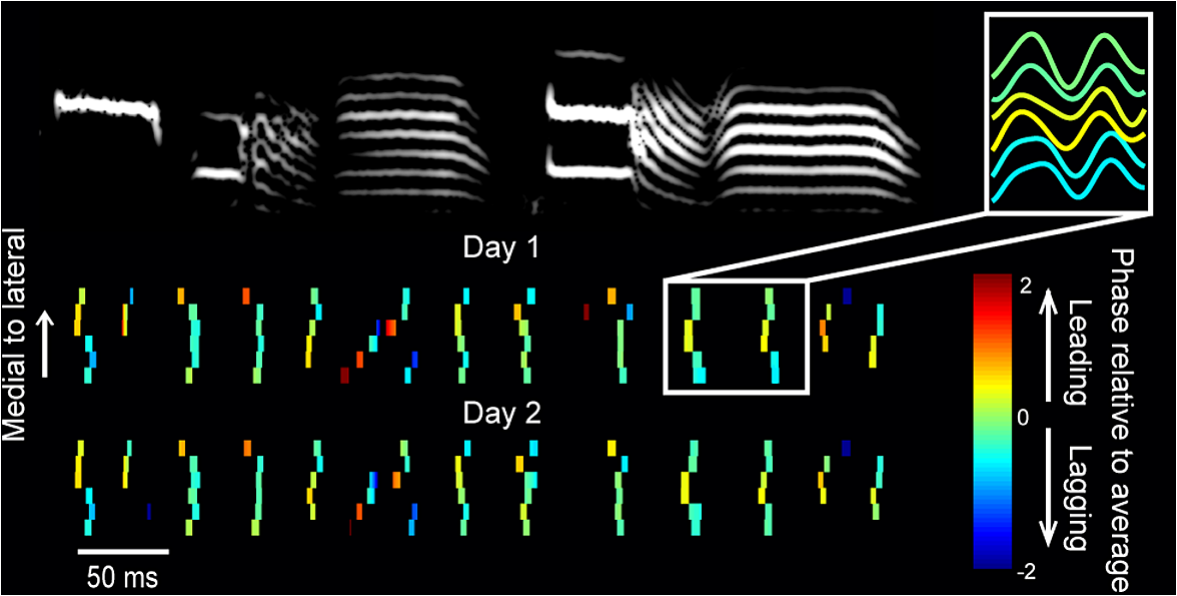
A stereotyped spatiotemporal 30 Hz pattern underlies premotor cortical activity during song. Trial-averaged 25–35 Hz LFP patterns on two consecutive days for six electrodes spaced by 175 μm along the mediolateral axis of HVC. Each tick mark indicates the timing of the local LFP peak, and the color indicates the phase relative to the average phase of all electrodes at that time point. (See breakout illustration top right.) See also **S4 Fig.**

**Fig 6:**
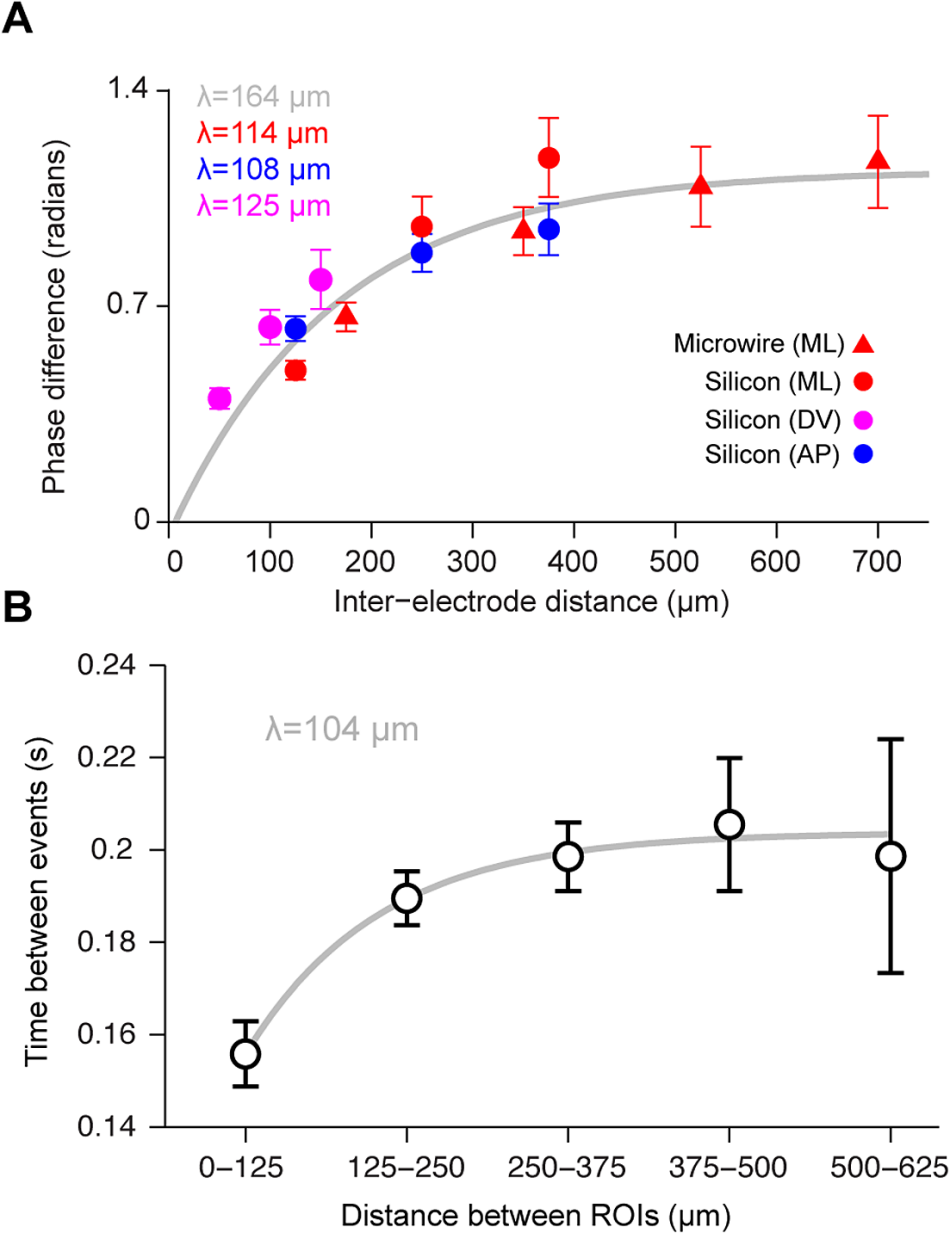
LFPs in the 25–35 Hz band and calcium events are correlated over a 100 μm length scale. **a**, Shown is the average phase difference between LFPs recorded on two separate electrodes as a function of distance along the dorsoventral (DV) (*n* = 70), mediolateral (ML) (*n* = 57), and anterior-posterior (AP) axes (*n* = 48). This data was collected using four-shank silicon probes (Neuronexus) and commercial microwire probes (TDT). **b**, Average difference in event times between ROIs observed using calcium imaging. The equation *M*(1 − exp(−*d*/*λ*))+*C* was fit to data in both **a** and **b** using a least squares procedure. In **a**, the data from each of the axes was separately fit (matching colors); the gray line represents the fit using data combined from the AP and ML axes. Error bars represent standard error of the mean (SEM). See also **S5 Fig.**

To characterize the length scale of the LFP correlation across the surface of HVC, we first implanted four bundles of four carbon fiber electrodes each (*n* = 5 birds), separated by approximately 200 μm in both the rostrocaudal and mediolateral axes of HVC. LFPs recorded from electrodes in the same bundle were highly similar, while those from different bundles revealed phases that were shifted by as much as 180° at some points in song (**S4B Fig**; *p* = 5.2e-11, z = -6.57, two-tailed Wilcoxon rank sum test). We also examined the spatial extent of LFP correlations using silicon and microwire arrays with larger spacing and well-defined geometries (see **Materials and Methods** for details). **Fig 5** reveals the details of the spatiotemporal LFP pattern for one bird, measured at six points along the mediolateral axis of HVC. The syllable-specific microstructure in the LFP phase is not just noise—the structure can be seen to repeat precisely from one day to the next as the bird sings his stereotyped song. Averaging over all recording sites in all birds, we found that the 25–35 Hz LFP has a length scale of 108–125 μm along the dorsoventral, mediolateral, and anterior-posterior axes (exponential fit to the difference in phase as a function of distance, **Fig 6A**). This LFP correlation length is comparable to that reported in other LFP studies [35,36] and calcium imaging studies of motor cortical activity [27,29,30], and approximately matches the correlation length of the calcium activity in projection neurons described above (exponential fit to the difference in calcium event times as a function of distance, **Fig 6B**). The LFP reflects a complex mixture of spiking and synaptic activity, but this analysis confirms through an independent modality that HVC activity is correlated over a 100 μm length scale during singing.

### The HVC LFP Reveals That, on Average, HVC_X_ and Interneurons Fire at Different Phases of a 25–35 Hz Inhibitory Rhythm

**Fig 7:**
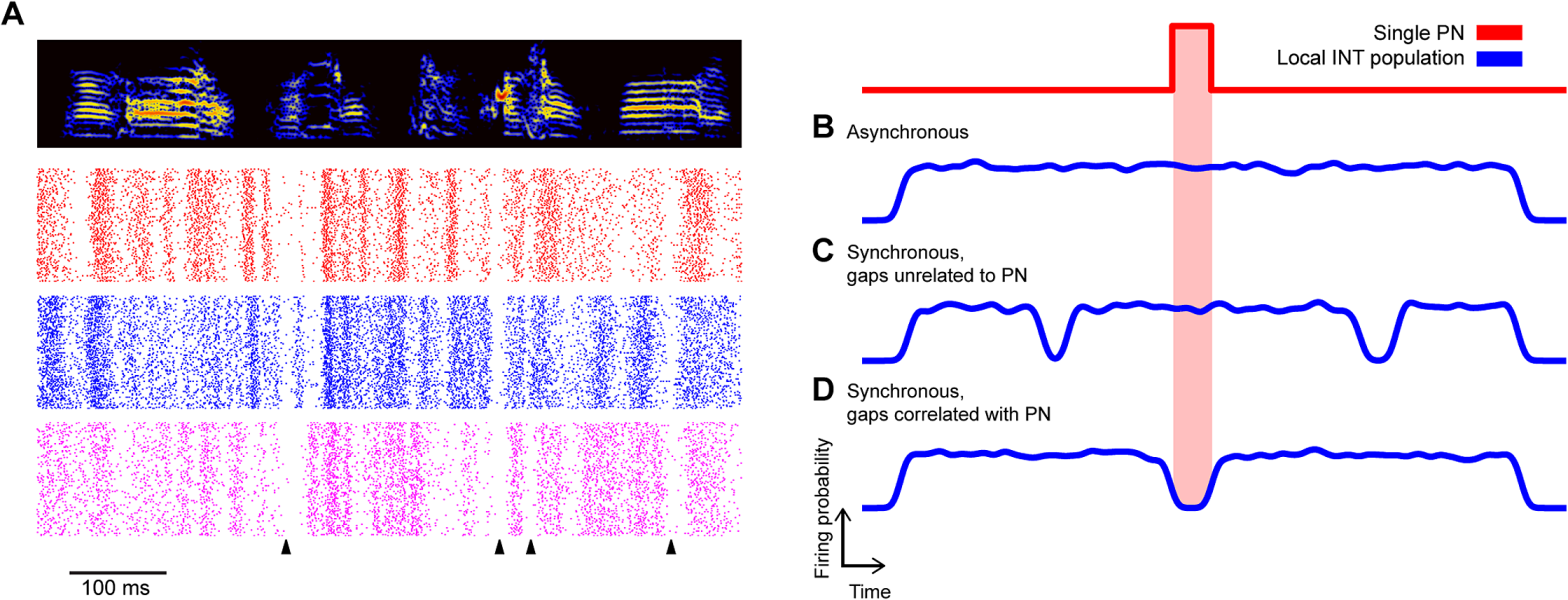
Inhibitory neuron firing patterns and three models for projection neuron/inhibitory neuron relationships. **a,** Raster plots of three nearby (<200 μm) inhibitory neurons recorded during singing from the same bird. Synchronous pauses are marked with a black triangle. Shown to the right are three conceptual models of timing relationships between inhibitory and projection neuron activity. **b,** Interneuron firing patterns are uncorrelated in local ensembles, leading to a net local inhibition that is weakly modulated in time. **c,** Gaps in inhibitory neuron firing activity are locally correlated, leading to synchronous pauses in inhibition, but these pauses are unrelated to projection neuron activity. **d,** Gaps in inhibitory neuron firing activity are locally correlated, and contribute to defining projection neuron firing times. PN, projection neuron; INT, interneuron.

**Fig 8:**
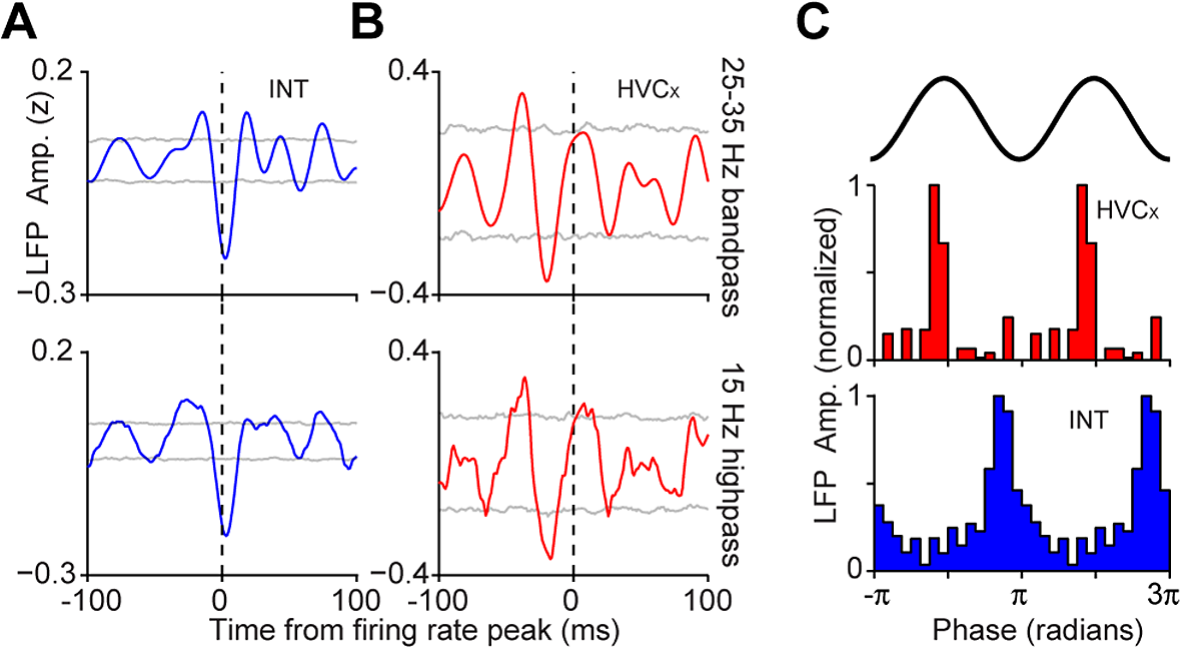
Spike-field analysis reveals an alternating 30 Hz rhythm in excitatory and inhibitory cells. **a,** Burst-triggered averages of 25–35 Hz (top row) and broadband LFPs (bottom) for interneurons (*n* = 65, *p* = 0, bootstrap test, see **Materials and Methods**) and (**b**) HVC_X_ neurons (*n* = 12, *p* =. 006, bootstrap test). Gray line indicates *p* =. 05. **c,** Here, the power of the LFP is binned at the phase of the LFP for each burst time. A clear separation in preferred phase can be seen between HVC_X_ neurons and interneurons.

We next sought to examine the relationship between interneuron and projection neuron timing. Projection neuron calcium transients are correlated over a 100 μm length scale. Similarly, LFPs, which are highly correlated with the timing of inhibitory neurons, are also correlated over a 100 μm length scale (**Fig 6A**). Previous work has shown that dense, stereotyped interneuron firing patterns occur during song (**Fig 7A**) [11,26], yet little is known about the relationship between projection neuron timing and patterned inhibition in singing birds. **Fig 7B–7D** illustrates three distinct possibilities: (1) local interneurons are asynchronous and population activity is not deeply modulated during song (i.e., there is no spatial structure, **Fig 7B**); (2) local interneurons show correlated peaks or troughs in firing rates, but these modulations are unrelated to projection neuron activity (**Fig 7C**); or (3) local interneurons manifest correlated peaks or troughs in firing rates, and these modulations are involved in defining excitatory projection neuron firing times (**Fig 7D**). While it was not possible to distinguish between these models by direct measurement of connected ensembles of identified cells, we can do this indirectly by analyzing cell-type specific firing rules in HVC in relation to the LFP. In other species and contexts, spike-field correlations have revealed information about the relative timing of excitatory and inhibitory cells in local volumes of the brain [38,41,42]. Here, to study the relationship between different cell types and the LFP, we computed burst-triggered averages of the LFP. For interneurons, the burst-triggered average reveals a trough in the LFP at approximately zero latency (**Fig 8A**). In contrast, HVC_X_ neurons (*n* = 12 cells, identified by the presence of two or more sparse bursts per song motif [26]) fire during peaks in the 25–35 Hz LFP (**Fig 8B**). The time-lag between HVC_X_ neurons and interneurons is particularly visible by binning the LFP power by the LFP phase at each burst time (**Fig 8C**)**,** revealing that HVC_X_ neurons fire in the peaks of the LFP, while interneurons fire in the troughs. (The remaining projection neurons, *n* = 7, fired only a single burst and could not be uniquely identified as either HVC_X_ or HVC_RA_ neurons and, so, were not included here.)

These observations indicate that the phase of the 30 Hz LFP is highly correlated with firing times in two cell classes in HVC, and suggest that HVC_X_ neurons, on average, fire during gaps in local inhibition. Anecdotally, within 16 channel carbon fiber bundles localized to volumes of 200 μm, we have observed multiple interneurons that produce correlated firing patterns and occasional synchronous pauses in inhibition across cells (**Fig 7A**). More rigorously, we find that interneuron troughs that occur within song syllables are more synchronous than one would expect by chance (median correlation coefficient .3848, .2895–.4652 99% bootstrap confidence interval; *p* = 0, bootstrap test, *n* = 71 interneuron pairs, see **Materials and Methods**). This analysis was restricted to periods of time when the bird was actively producing sound. As a result, this analysis is not dominated by any modulations in firing rates related to the pattern of sound and silence that occurs within a song motif. Since the precise locations of the carbon fiber electrode tips were not known, we could not relate the degree of correlation to the spatial separation of interneurons. Synchronous pauses in inhibition could be responsible for the dips in the LFP, and could also provide a “window of opportunity” for projection neurons to fire. At least one class of projection neuron, the HVC_X_ neuron, tends to fire out of phase with inhibition in a 30 Hz timescale.

## Discussion

In singing birds, the relationship between time and space in premotor cortical area HVC is neither random, nor globally organized as a traveling wave, but clustered on a 100 μm length scale. A 30 Hz rhythm, revealed in the LFP and correlated with inhibition, influences the timing of at least one projection neuron sub-type (HVC_X_), and this rhythm is coherent over a length scale matching that of projection neuron calcium activity (**Fig 6**).

The mesoscopic pattern apparent in the LFP and calcium imaging has not been observable using prior methods of sampling single cell activity serially. The stereotyped behavior of the songbird has allowed for alignment of multiple renditions of song and the construction of “pseudo-ensembles” based on populations of cells that were not simultaneously recorded [26]. This approach has proven to be powerful in the song system and has led to rapid advances in understanding the song circuit, but it is an approach that cannot resolve microcircuit dynamics in small volumes of HVC and cannot resolve spatial maps on the length scale described here. For lack of experimental data, prior dynamical models of HVC have not considered local spatial correlations in time cell firing [6]. Most models also ignore the contribution of inhibitory interneurons [12] or assume that inhibition guards against runaway excitation [6] but is otherwise uninvolved in shaping projection neuron firing times (though there are a few notable exceptions [20]).

In contrast, we find that the local microcircuit patterns in HVC in singing birds include correlations over space and specific rules for the relative timing of distinct cell types. Our results reveal that HVC contains “time domains” characterized by nearly synchronous projection neuron calcium activity. The 100 μm correlation length of the projection neurons also appears in the 30 Hz LFP, a signal that is highly correlated with an inhibitory rhythm expressed locally in HVC (**Fig 4B and 4C** and **Fig 8A**). Within a local patch of HVC, the LFP and spike correlations indicate that, on average, HVC_X_ projection neurons fire in gaps of inhibition (**Fig 8B**). Taken together, these results indicate that functional domains within the HVC microcircuit may contain tightly correlated populations of excitatory and inhibitory cells that fire in opposition and generate a local 30 Hz rhythm in the LFP. These “time domains” resemble the intermediate scale of geometric organization illustrated in **Fig 1B**, characterized by zones in which projection neurons fire at similar times with no coherent global organization.

Important questions remain about the microcircuit dynamics of HVC. Some are technical; the imaging results reported here rest on calcium imaging, and the relationship between calcium transients and the underlying burst of action potentials in HVC projection neurons has not been measured during singing. At present, we can say that to first approximation, the sparse calcium transients match the expected burst behavior of the excitatory cells in HVC. The head-mounted microscope provided only single-color imaging, precluding cell type identification based on retrograde labeling of downstream targets. Similarly, the rhythm between HVC_X_ cells and interneurons is inferred based on average timing relative to a common signal rather than direct measurements of connected pairs of cells. Our electrophysiology did not address the relationship between HVC_RA_ projection neurons and the inhibitory rhythm. The role each cell type in HVC plays in driving or responding to the local inhibitory rhythm, and the connectivity that gives rise to this rhythm, await more detailed description [22,23]. Moreover, neither the imaging nor the electrophysiology could directly measure the correlation length of the inhibitory neurons. This was extrapolated from the de-phasing length scale of the 30 Hz LFP—a measure clearly related to interneuron firing patterns, but still an imperfect surrogate.

Perhaps more interesting are the outstanding questions about the nature of a dynamical system governed by the properties observed here. Inhibitory neurons show patterned activity throughout song, whereas projection neurons fire sparsely, with calcium events that are correlated in time between neighbors. This suggests a microcircuit in which multiple pools of excitatory neurons drive local interneuron populations. Then, in a feedback loop, locally synchronized inhibition on a 30 ms timescale also influences the firing times of multiple pools of excitatory neurons. Theoretical models of neural sequence generation have focused on the direct interactions between excitatory neurons, but our results suggest that the indirect interactions between excitatory neurons, mediated through inhibition, may be important in defining which cells fire at a given time. In this view, the timing of a projection neuron’s activity may be defined not only by its connections to other projection neurons but also by rhythmic patterns of inhibition dominated by a 30 Hz timescale. Considering the importance of excitatory-inhibitory coupling in defining the timing of at least one class of projection neuron in HVC, the space of possible models for HVC activity expands significantly.

HVC is one stage in a motor cortico-thalamic loop, and intriguingly, the timescale of activity propagation around this loop is similar to the 30 Hz rhythm described here [43]. Future work is needed to examine whether this timescale is intrinsic to cellular biophysics in HVC or arises from the time it takes excitatory pulses to propagate around the cortico-thalamic loop [44]. Whether this 30 ms timescale is related to acoustic transitions in song remains to be examined [45,46] and will require more extensive sampling of excitatory and inhibitory cells over the entire volume of HVC.

Functional clustering in HVC has been described previously in the spontaneous activity of anesthetized songbirds [15], but we note a possible difference in the length scale reported in the spontaneous activity of anesthetized birds (λ = 263 μm) and the length scale we observe in singing birds (λ = 104 μm for calcium imaging, λ = 108–125 μm for LFPs). The length scale of correlation during singing matches functional clustering on a 100 μm length scale that has been observed in mammalian motor cortex in the context of running, grooming [27], and simple forelimb movements [29,30]. Additionally, the 30 Hz rhythm has parallels in primate motor control [28,31,32] and speech perception and production in humans [47]. The recurrence of these length scales and rhythmic timescales across species and contexts raises the possibility that spatial clustering and 30 Hz rhythmic alternation of excitatory and inhibitory cells may be a recurring feature of cortical motor microcircuits and critical for the sequential organization of behavior. If true, this would imply that relatively slow movements produced by primates and the fast vocal movements of a songbird are coarse-grained in a similar fundamental length scale (100 μm) and timescale (30 Hz) at the level of cortical control.

For studies of songbird vocal learning, these results suggest that future biophysical models of motor sequence generation in HVC should move away from abstract models of neuronal chains defined only by the topology of excitatory cells, to consider new classes of models in which the interplay of spatially correlated patterns of excitation and inhibition form the basis of the stereotyped, robust sequences of neural activity driving birdsong.

## Materials and Methods

### Subjects

All procedures were approved by the Institutional Animal Care and Use Committee of Boston University (protocol numbers 09–007 and 11–027). Electrophysiology data were collected from 27 adult male zebra finches (>120 DPH), and imaging data were collected from nine adult male zebra finches. Birds were kept on a 14 h light-dark cycle.

### Viral Labeling and Calcium Imaging

Chronic calcium imaging was performed using a head-mounted fluorescent microscope described in detail elsewhere [48]. To label HVC neurons with GCaMP6s calcium indicator, birds received four 250 nl injections of lentivirus packaged with GCaMP6s under a Rous sarcoma virus (RSV) promoter into HVC [49]. To guide the injection of virus, the bounds of HVC were determined by bipolar antidromic stimulation of Area X (*n* = 3) or through fluorescence imaging of a DiI retrograde tracer (*n* = 6) [18]. We collected 167 ROIs from nine birds. Fluorescent traces for each animal were collected over a single day of imaging. For the calcium imaging, all songs were directed (i.e., a female was present).

Our unpublished results indicate that the RSV promoter in HVC does not label interneurons or glial cells. We have infected HVC with viruses with the RSV promoter driving transcription of several genes fused to GFP and performed double immunocytochemistry against GFP and several markers for inhibitory interneurons (parvalbumin, somatostatin, and calretinin). We did not observe co-localization of cells labeled with GFP with any of the interneuronal markers. In addition, we also observed that the morphology of the GFP-positive cells was consistent with that previously described for projection neurons in HVC, but not consistent with them being glial cells. This observation suggests that the RSV promoter labels projection neurons in HVC.

GFP immunolabeling and confocal imaging of the RSV-GCaMP6s construct used here revealed that in all cases in which dendrites were visible, they contained spines, consistent with the morphology of HVC_RA_ and HVC_X_ projection neurons. Thirty percent of all infected neurons were HVC_X_ projection neurons, based on retrograde labeling from Area X. Retrograde labeling was not performed from RA, but GFP immunolabeling revealed dense axon arbors in nucleus RA, indicating that a significant number of HVC_RA_ neurons were also labelled.

### Electrophysiology

Electrophysiological recordings were gathered from 27 birds, in which 13 received implants from 16-channel carbon fiber bundles, five from four four-channel carbon fiber bundles, three from tungsten microwire arrays (Tucker Davis Technologies, 16-channel 33 μm wire diameter), and six from four-shank silicon probes (*n* = 4 along the mediolateral axis, *n* = 2 along the anterior-posterior, Neuronexus model no. A4x4-3mm-50-125-703-CM16, 15 μm thickness). All arrays were implanted. 7 mm anterior and 2.3 mm lateral of the midsagittal sinus at a depth of. 4–.7 mm (head angle 30°) using previously described methods [11]. All songs used for electrophysiology were undirected (i.e., no female was present). In a subset of the implants, the position of the implant (but not single units) was verified antidromically with bipolar stimulating electrodes placed in Area X [3]. Excitatory projection neurons and putative interneurons were identified based on their unique firing properties. In previous studies, antidromically identified HVC_X_ projection neurons were shown to produce two or more sparse, time-locked bursts per motif. Putative interneurons produced dense, tonically active, time-locked firing patterns [3]. All neurons reported in this study fell into one of these two categories. Local field potentials (LFPs) were extracted from the same electrodes used to record single units after spike removal, and all statements about spike-field relationships were also confirmed with an analysis in which the LFPs were extracted from nearby electrodes, indicating that no cross-contamination between spikes and fields influenced these results.

### Analysis of Calcium Imaging Data

All offline analyses of the calcium imaging data were performed using custom software written in MATLAB. First, the raw imaging data was motion corrected using a previously published algorithm [50]. Regions of interest (ROIs) were then manually selected and converted into single cell fluorescence traces by taking the mean intensity of all pixels within a given ROI for each movie frame. Baseline fluorescence for each trace, F_0_, was computed by taking the 12th percentile in an 800 ms sliding window (±400 ms at each time point). Changes in fluorescence were then defined as ΔF/F_0_ = (F−F_0_)/F_0_, where F is the fluorescence of a given ROI and a particular point in time. Event times were computed by first finding all local peaks in the ΔF/F_0_ trace that exceeded 1%. Events were excluded if ΔF/F_0_ did not remain above -1% for 300 ms after the detected peak. Then, the event time was defined as the 50% rise time between the peak and the preceding trough [48]. To provide an upper bound on the false positive rate given these criteria, which were chosen manually based on visual inspection of the data, we computed the likelihood of finding a peak when the birds were not singing (*p* =. 0156 from 61.5 seconds of silence). All spatial correlations were computed using these event times, derived from temporally sparse fluorescence transients in spatially sparse populations of infected neurons. The correlation length scales calculated from these event times are robust to any out of focus fluorescence or neuropil signal that could add spurious correlations to the calcium time series.

We also note that the distance measurements used for the spatial correlations are exact only if the imaged cells are all in the same plane, which is not the case here. The depth of each cell could not be determined since the head-mounted cameras were used in a fixed location. To estimate the error due to this ambiguity, we focused the microscope sharply on 1 μm beads in agarose and then defocused until the image of a bead expanded to the size of the largest selected ROI. This provides a conservative measurement of how far outside the imaging plane a cell can be while still meeting the selection criteria of an ROI. A more realistic measure was computed by defocusing from a spot the same size as the smallest ROI until it was distorted the same size as the largest ROI. By the conservative measure, cells that appear overlapping in the XY plane may be separated by a *z*-axis distance of 35 μm (22 μm by the more realistic measure). However, the true ambiguity in the Euclidian cell-to-cell distance, taking into account the ambiguity in the Z plane, will decrease as ROIs appear more distant in the XY plane. Cells observed to be 50 μm apart will have an uncertainty of 11 μm (4.5 μm); at 100 μm, the uncertainty is only 6 μm (2.4 μm).

### Bootstrap Tests of Imaging Data

The analyses shown in **Fig 2F and 2G** and **S1B Fig** were all conducted using randomization tests. For **Fig 2F**, the median distance between ROIs was plotted against the time between their respective calcium events. To test for significance at each time bin, the same number of ROIs were randomly selected from the entire dataset 10,000 times, and the median was computed for each randomization. The *p*-value is then the probability that a median value from the randomized distribution is lower than the observed median. In **Fig 2G**, the same procedure was used to test the length of the resultant vector. That is, we tested whether the length of the resultant vector was longer than would be expected using the same randomization. Finally, for **S1B Fig**, we used a similar procedure to test for the significance of the standard deviation of the anterior-posterior and mediolateral distance distributions.

### Analysis of Electrophysiology Data

Extracellular voltage traces were multiplexed and digitized at 25 kHz on the headstage and transferred to a PC over USB (Intan RHA2000). Song was recorded using a head-mounted microphone glued to the headstage [11]. All offline analyses were performed using custom software written in MATLAB (Mathworks). To isolate single units offline, the extracellular voltage traces were bandpass filtered between 600 and 11,000 Hz (12th order Elliptic filer, .2 dB passband ripple, 40 dB stopband attenuation) and sorted using standard offline spike sorting techniques [51,52]. For the LFPs, voltage traces were first median filtered (1 ms window) to remove spikes [53], then lowpass filtered with a 400 Hz corner frequency (4th order Butterworth filter) and downsampled to 1 kHz. To isolate the 25–35 Hz LFP, the downsampled LFP was bandpass filtered between 25–35 Hz (149 tap Kaiser window FIR filter, 40 dB stopband attenuation, .05 ripple). To minimize the impulse response, a more lenient 25–35 Hz bandpass was used for the spike-triggered analysis shown in **Fig 8A and 8B (top)** and for the comparison shown in **Fig 4C** (53 tap Kaiser window FIR filter, 20 dB stopband attenuation, .05 ripple). All event-triggered LFP analyses were also repeated using only a 15 Hz highpass filter (**Fig 8A and 8B bottom**; 84 tap Kaiser window FIR filter, 20 dB stopband attenuation, .5 ripple). All filtering was performed forwards and backwards to correct for phase distortion. Songs were aligned using previously described methods [54]. Spike-field coherence was estimated using standard techniques based on the cross-spectrum between the spike train and the LFP [55] (1 Slepian taper, NW = 1). To control for bias, the same number of trials were used for each neuron. Additionally, we only included high-quality single units for measuring spike field coherence (*n* = 17 interneurons, SNR > 8 and <.1% ISIs <1 ms, no sign of cluster contamination), all other measures included all interneurons.

### Song Alignments

Songs were aligned using previously described methods [54]. In brief, trials were aligned to song by computing the Euclidean distance in spectral features between the data and a template song in a sliding window. Troughs in the distance were considered candidate matches, and the spectral features of all potential hits were plotted in 2 dimensions and the user performed a cluster cut.

### Spike Sorting

First, positive- and negative-going threshold crossings in the 600–11,000 Hz bandpassed extracellular voltage traces were detected. The threshold was set to four times the bandpassed signal’s robust standard deviation [56]. Then, a 1.1 ms window centered on the negative peak was saved. The spikes were then re-aligned to the negative peak after upsampling by a factor of 8 using cubic splines. Features of the aligned spike windows were computed using robust principal components analysis [51]. A mixture of Gaussians model was fit to the spike features using the split and merge expectation maximization algorithm [52], and the number of components in the mixture was determined by fitting models with 2–7 components and choosing the model with the minimum Bayes Information Criterion [52]. Unit quality was then assessed by signal-to-noise ratio and refractory period violations. For multi-unit analysis (**S4B Fig**) all threshold crossings above three standard deviations of the bandpassed signal were included.

### Spectral Density Images

To compute the spectral density image for multiple aligned renditions of the same LFP, we used previously described methods [40]. First, a sparse, binary time-frequency representation of each rendition was generated using “auditory contours” [57]. Then, the binary representations were combined by summing across all renditions. In this representation, a false-color plot (**Fig 3A** bottom) represents the probability of a binary contour passing through a given point in the time-frequency plane.

### Identifying Bursts in Projection Neurons and Interneurons for Burst-Triggered Averaging

To identify bursts of single-unit activity, we first estimated the smooth firing rate for each trial using a Gaussian kernel (*σ* = 5 ms). The smooth firing rate was averaged across trials, and we counted peaks above 100 Hz as burst events.

### Computing Firing Pattern Distance

We define the distance between two firing patterns as the mean absolute difference between their average firing rates. The firing rate was estimated by computing the smooth firing rate for each trial (Gaussian kernel, *σ* = 5 ms), and then averaging across trials.

### Determining Whether Interneuron Troughs Are Synchronous

To determine whether interneuron troughs within syllables were more coherent than one would expect by chance, we conducted a bootstrap procedure. First, the firing rate was estimated within syllables by computing the smooth firing rate for each trial (Gaussian kernel, *σ* = 5 ms), averaging across trials, and z-scoring the average. All negative-going peaks less than −.5 were counted as troughs. A binary vector was created, with 1’s indicating the presence of a trough and 0’s denoting all other time points (troughs in inter-syllable gaps were excluded), and each vector was smoothed with a Gaussian kernel (*σ* = 5 ms). Then we computed the Pearson correlation coefficient between these smoothed vectors from all pairs of interneurons from the same bird (carbon implants only, *n* = 71 pairs). The median correlation coefficient across all birds was then compared to a null distribution estimated using a bootstrap procedure in which the troughs were selected randomly from time points during syllables (*n* = 1,000 bootstraps).

### Phase Locking

All phase locking indices (PLIs) were computed using previously described methods [58].

#### Local phase locking across frequencies

To compute the PLI on a single electrode across trials in multiple frequency bands (PLI_STFT_, **Fig 4A**), we first extracted the phase angle for each point in time and frequency from the broadband downsampled LFP (0–400 Hz) using the angle of the complex STFT (125 ms Hanning window, 100 ms steps). At each point in time and frequency, the local phase locking index is defined as ${\text{PLI}}_{\text{STFT}}=\frac{1}{N}\left|\sum_{n=1}^{N}\exp(j\phi (t,f,n))\right|$, where *ϕ*(*t*,*f*,*n*) is the phase at each point in time *t*, frequency bin *f*, and trial *n*. For the comparison shown in **Fig 4A**, we computed the average baseline Rayleigh statistic (see below) using the 200 ms silent period before and after song, and subtracted it from the average Rayleigh statistic computed during singing.

#### Local phase locking in the 25–35 Hz band

To compute the PLI on a single electrode across trials within the 25–35 Hz band (PLI, **S3 Fig**), we first computed the phase at each point in time using the angle of the Hilbert transform of the 25–35 Hz filtered LFP. Then, we computed $\text{PLI}=\frac{1}{N}\left|\sum_{n=1}^{N}\exp(j\phi (t,n))\right|$, where *ϕ*(*t*,*n*) is the phase at each point in time *t* and trial *n*.

#### Rayleigh statistic

PLI values are expressed as the standard Rayleigh statistic, Z = R^2^ / *n*, where R is Rayleigh’s R, R = *n*PLI, and *n* is the number of trials [59]. *P*-values were then estimated using the following equation, $P=\exp\left[\sqrt{1+4n+4(n^{2}-{\text{R}}^{2})-(1+2n)}\right]$ [60].

### Statistical Tests

No formal methods were used to predetermine sample sizes, but the sample sizes used here are similar to those generally used in the field. All statistical comparisons were performed using either a one-tailed or two-tailed Wilcoxon ranksum test, with four exceptions. First, the significance of the imaging results was determined using a bootstrap procedure (**Fig 2F and 2G** and **S1B Fig**; described above). Second, the significance of spike-field coherence (**Fig 4B**) was determined using a bootstrap procedure by shifting the spike trains across all trials by a separate random number for each neuron (1,000 bootstraps). Third, the significance of burst-triggered average LFPs was computed using a bootstrap procedure. Burst times were randomized for each neuron (1,000 bootstraps), and the maximum absolute value was compared between the observed burst-triggered average and the bootstraps. Finally, the statistical significance of the trough timing was determined using a bootstrap in which the troughs were randomly selected from time points during syllables (1,000 bootstraps). When appropriate, we controlled for multiple comparisons using the Bonferroni correction.

## Supporting Information

S1 Fig Coactive ROIs are significantly clustered along the anterior-posterior axis. **a,** Relative distances between all coactive ROIs (<20 ms between calcium events, *n* = 104 ROI pairs). **b,** Distributions of relative distances along the mediolateral and anterior-posterior axes (mediolateral in red, anterior-posterior in blue). The standard deviation of the mediolateral distribution is significantly smaller than would be expected according to a random model in which event times are randomly distributed throughout the nucleus (*p* = 2e-4, bootstrap test; *p* =. 1961 for the anterior-posterior distribution). Neither axis has a significantly larger standard deviation (*p* = 1 for the mediolateral distribution, *p* =. 8067 for the anterior-posterior).
(TIF)

S2 Fig LFP single trial examples. Broadband and filtered LFPs, showing trial-averaged LFPs and a random sample of single-trial traces. *Top*, The LFP is bandpass filtered from 5–200 Hz (6th-order Elliptic filter, .2 dB passband ripple, 40 dB stopband attenuation). *Bottom*, LFP bandpass filtered from 25–35 Hz, same trials and format as top.
(TIF)

S3 Fig Analysis of phase stereotypy in the 25–35 Hz LFP. **a,** An example of the trial-averaged LFP power in red, PLI in blue, and LFP envelope in green. Power and envelope are defined as follows: LFP power = ⟨|H(LFP)|^2^⟩, where H indicates the Hilbert transform, LFP the 25-35Hz LFP, and ⟨ ⟩ the trial average over songs; LFP envelope = |H(⟨LFP⟩)|. In this example, the temporal modulation of the envelope correlates with PLI but not with power. This trend is shown for the full dataset in the next panel. **b,** For the population, the envelope is highly correlated with PLI, and less correlated with power. **c,** LFP phase consistency (PLI) is modulated by the pattern of sound and silence in song. PLI is highest during syllables (*n* = 2339 bins, values were first averaged in 30 ms non-overlapping bins), lower during inter-syllable gaps (*n* = 531 bins, *p* = 2e-7, z = 5.4), and much reduced in the 200 ms period before (*n* = 539 bins, *p* = 9.1e-53, z = 15.36) or after song (*n* = 726 bins, *p* = 3.2e-80, z = 19.02) (***, *p* <. 001 two-tailed Wilcoxon ranksum test, Bonferonni corrected). Error bars indicate SEM. The actual drop in PLI during gaps could be much lower, as PLI is smoothed by the 75 ms timescale of the 25–35 Hz LFP filter. (The typical gap duration for zebra finches is 50 ms [61].)
(TIF)

S4 Fig Local correlations in 25–35 Hz LFPs and multi-unit activity. **a,** Trial-averaged LFPs from three four-wire bundles (wires within a bundle are spread over 25 μm zones, and grouped together in this figure. The typical distance between bundles was 200 μm). **b,** Multi-unit activity (*n* = 331 channel pairs, *p* = 3.1e-5, z = 4.16) and LFPs (*n* = 527 channel pairs, *p* = 5.2e-11, z = -6.57) are more similar on nearby electrodes than on distant electrodes (***, *p* <. 001, two-tailed Wilcoxon ranksum test). Bars indicate mean and error bars SEM.
(TIF)

S5 Fig Detailed analysis of the spatial correlations of the 25–35 Hz LFP shown in Fig 6. **a,** The mean change in LFP phase across the dorsoventral, mediolateral, and anterior-posterior axes recorded using commercial silicon probes. **b,** The mean change in LFP phase across the mediolateral axis recorded using commercial microwire probes. Error bars indicate SEM. All tests were one-tailed Wilcoxon ranksum except for the pairwise comparisons between the AP and ML data, which were two-tailed. The Bonferonni correction was applied to all *p*-values.
(TIF)

S1 Movie Example of calcium activity during a complete song bout. Playback speed is 1x (22 frames per second).
(MP4)

## Abbreviations:

AP: anterior-posterior
DV: dorsoventral
LFP: local field potential
ML: mediolateral
PLI: phase locking index
ROI: region of interest
