# Supplementary figures and images for: Mesoscopic Patterns of Neural Activity Support Songbird Cortical Sequences
Source: PLoS Biol. 2015 Jun 3;13(6):e1002158. doi: 10.1371/journal.pbio.1002158 (PMC4454690; doi:10.1371/journal.pbio.1002158)

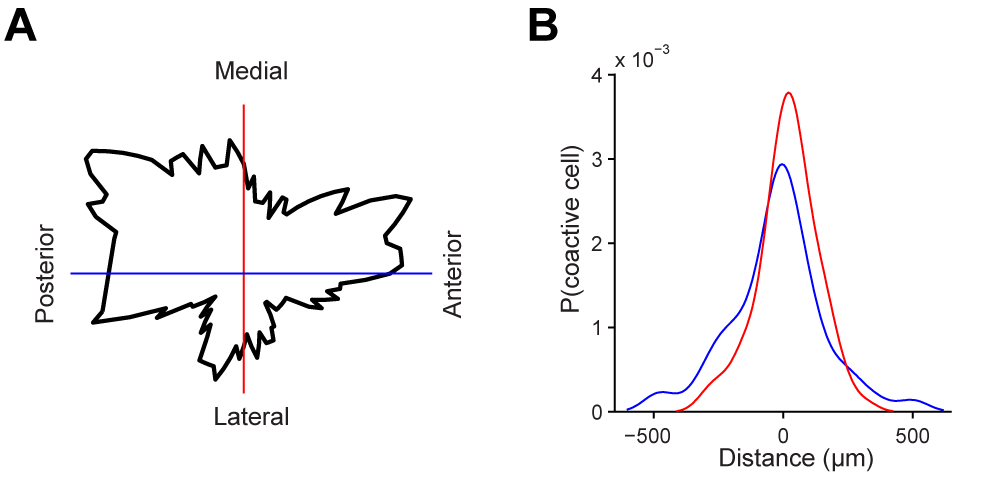

Supplement: S1 Fig — a, Relative distances between all coactive ROIs (<20 ms between calcium events, n = 104 ROI pairs). b, Distributions of relative distances along the mediolateral and anterior-posterior axes (mediolateral in red, anterior-posterior in blue). The standard deviation of the mediolateral distribution is significantly smaller than would be expected according to a random model in which event times are randomly distributed throughout the nucleus (p = 2e-4, bootstrap test; p =. 1961 for the anterior-posterior distribution). Neither axis has a significantly larger standard deviation (p = 1 for the mediolateral distribution, p =. 8067 for the anterior-posterior). (TIF) [file pbio.1002158.s001.tif]

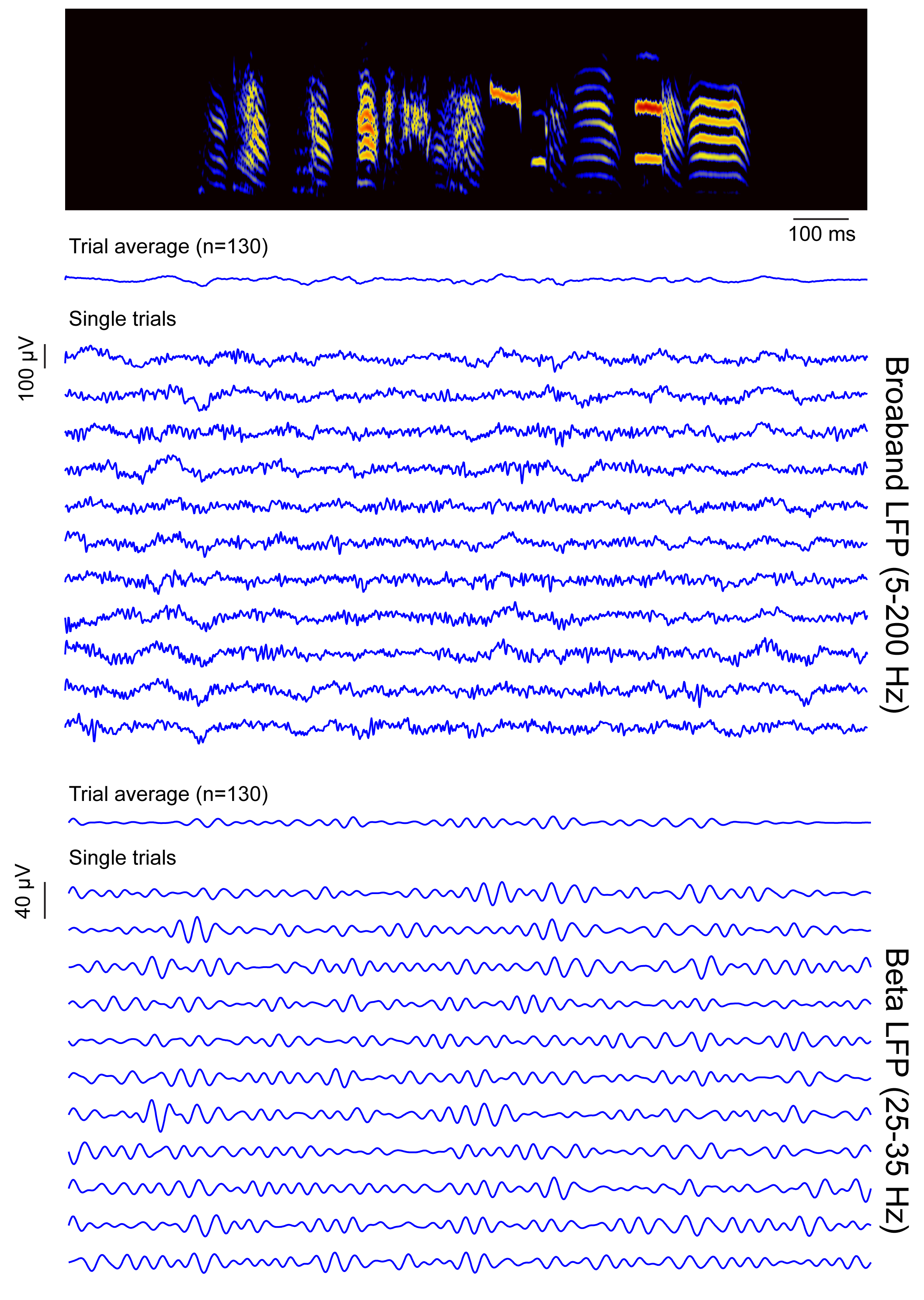

Supplement: S2 Fig — Broadband and filtered LFPs, showing trial-averaged LFPs and a random sample of single-trial traces. Top, The LFP is bandpass filtered from 5–200 Hz (6th-order Elliptic filter, .2 dB passband ripple, 40 dB stopband attenuation). Bottom, LFP bandpass filtered from 25–35 Hz, same trials and format as top. (TIF) [file pbio.1002158.s002.tif]

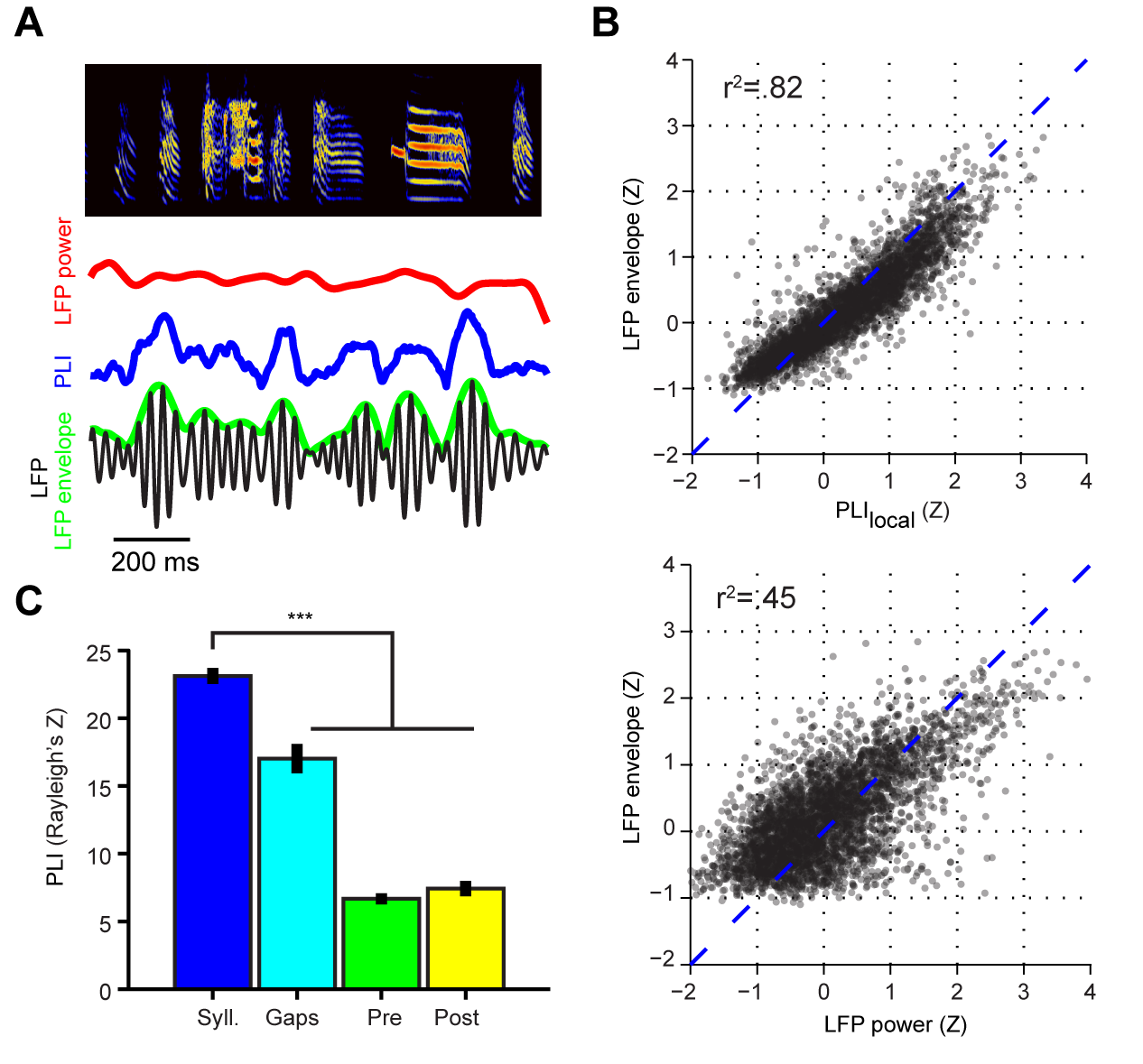

Supplement: S3 Fig — a, An example of the trial-averaged LFP power in red, PLI in blue, and LFP envelope in green. Power and envelope are defined as follows: LFP power = ⟨|H(LFP)|2⟩, where H indicates the Hilbert transform, LFP the 25-35Hz LFP, and ⟨ ⟩ the trial average over songs; LFP envelope = |H(⟨LFP⟩)|. In this example, the temporal modulation of the envelope correlates with PLI but not with power. This trend is shown for the full dataset in the next panel. b, For the population, the envelope is highly correlated with PLI, and less correlated with power. c, LFP phase consistency (PLI) is modulated by the pattern of sound and silence in song. PLI is highest during syllables (n = 2339 bins, values were first averaged in 30 ms non-overlapping bins), lower during inter-syllable gaps (n = 531 bins, p = 2e-7, z = 5.4), and much reduced in the 200 ms period before (n = 539 bins, p = 9.1e-53, z = 15.36) or after song (n = 726 bins, p = 3.2e-80, z = 19.02) (***, p <. 001 two-tailed Wilcoxon ranksum test, Bonferonni corrected). Error bars indicate SEM. The actual drop in PLI during gaps could be much lower, as PLI is smoothed by the 75 ms timescale of the 25–35 Hz LFP filter. (The typical gap duration for zebra finches is 50 ms [61].) (TIF) [file pbio.1002158.s003.tif]

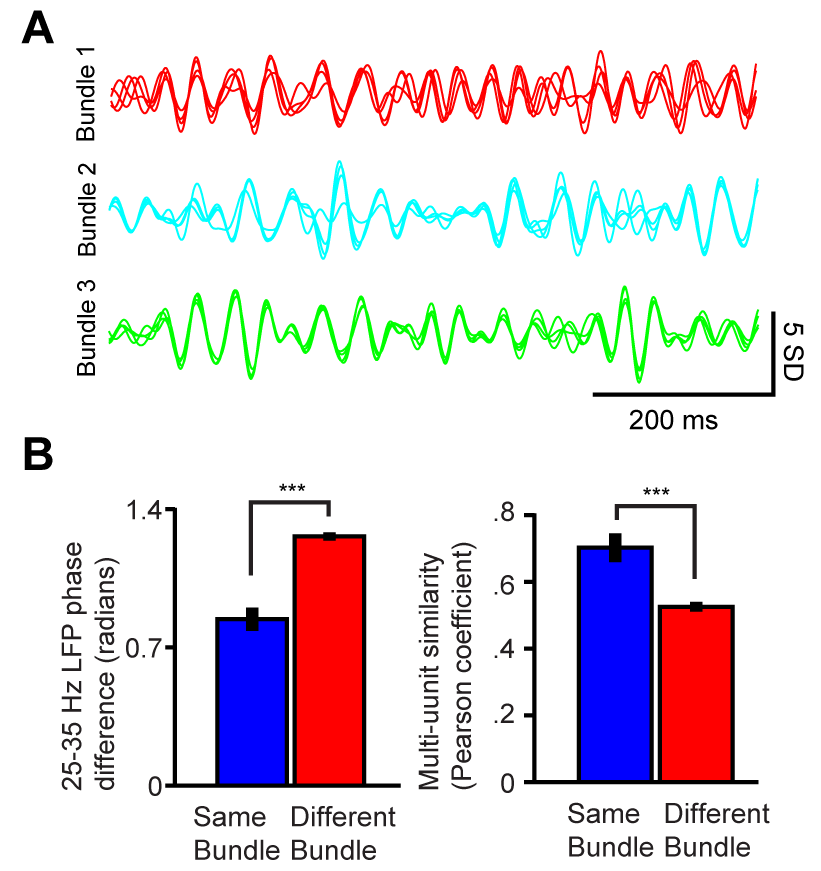

Supplement: S4 Fig — a, Trial-averaged LFPs from three four-wire bundles (wires within a bundle are spread over 25 μm zones, and grouped together in this figure. The typical distance between bundles was 200 μm). b, Multi-unit activity (n = 331 channel pairs, p = 3.1e-5, z = 4.16) and LFPs (n = 527 channel pairs, p = 5.2e-11, z = -6.57) are more similar on nearby electrodes than on distant electrodes (***, p <. 001, two-tailed Wilcoxon ranksum test). Bars indicate mean and error bars SEM. (TIF) [file pbio.1002158.s004.tif]

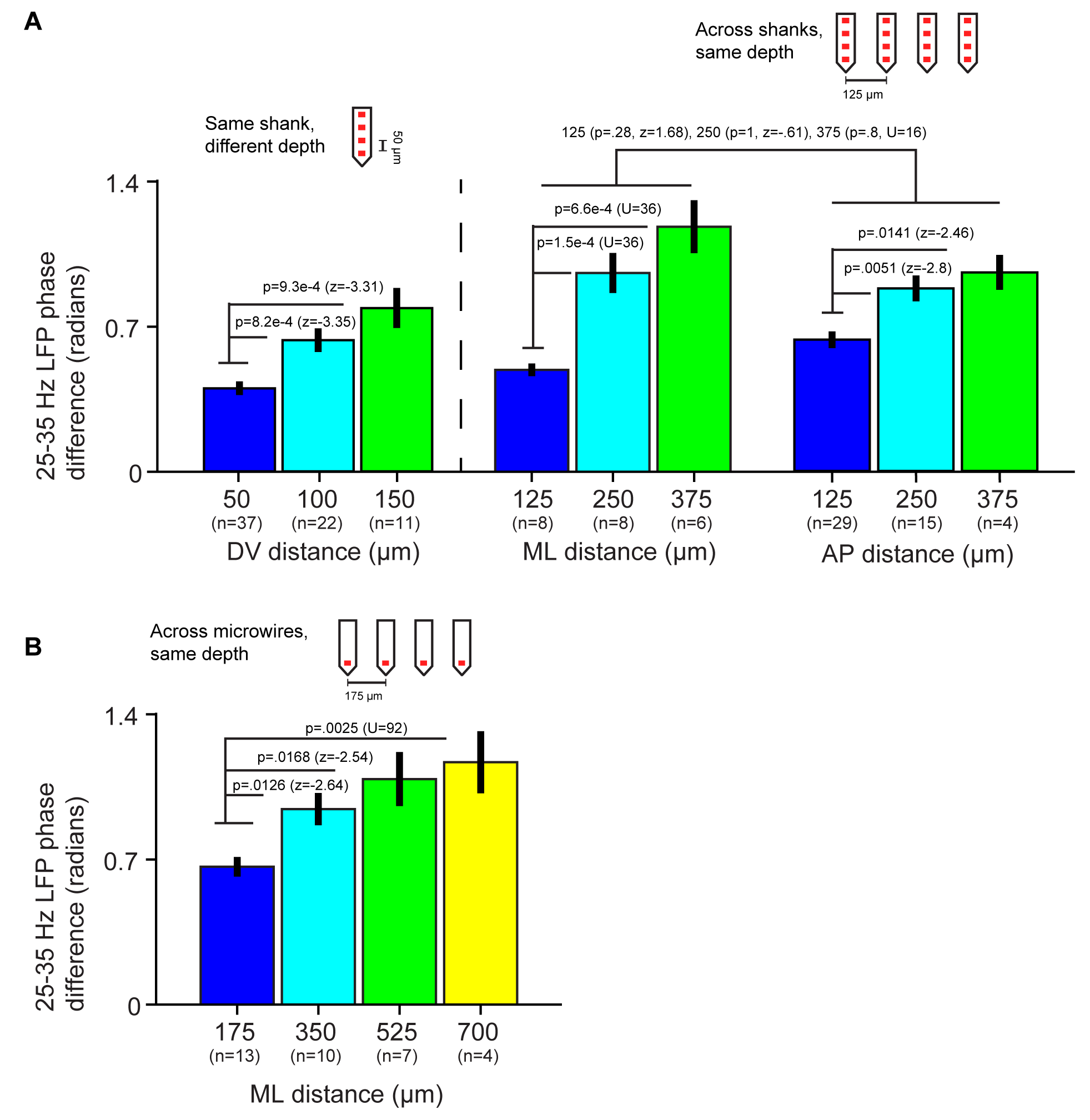

Supplement: S5 Fig — a, The mean change in LFP phase across the dorsoventral, mediolateral, and anterior-posterior axes recorded using commercial silicon probes. b, The mean change in LFP phase across the mediolateral axis recorded using commercial microwire probes. Error bars indicate SEM. All tests were one-tailed Wilcoxon ranksum except for the pairwise comparisons between the AP and ML data, which were two-tailed. The Bonferonni correction was applied to all p-values. (TIF) [file pbio.1002158.s005.tif]
